# The Effect of Virtual Reality Exercise Program on Sitting Balance Ability of Spinal Cord Injury Patients

**DOI:** 10.3390/healthcare9020183

**Published:** 2021-02-09

**Authors:** Min-Jae Lee, Sun-Min Lee

**Affiliations:** 1 Department of Occupational Therapy, Chungnam State University, Daejeon 33303, Korea; mjlee@cnsu.ac.kr; 2 Department of Occupational Therapy, College of Rehabilitation Science, Daegu University, Gyeongsan-Si 38453, Korea

**Keywords:** virtual reality system, spinal cord injury, sitting balance ability

## Abstract

(1) Background: Virtual reality (VR) is a useful device for rehabilitation therapy. The purpose of this study was to examine the effect of virtual reality exercise program on sitting balance with spinal cord injury; (2) Methods: 20 subjects who selected on the basis of the screening criteria were divided into the experimental group (*n* = 10) who underwent the virtual reality exercise program and rehabilitation therapy and the control group (*n* = 10) who underwent a regular sitting balance training program and a regular rehabilitation therapy. Each intervention consisted of a 30-min session a day, three times a week, for eight weeks. In order to measure functions of the sitting balance, FSA (force sensitive application) and LOS (limit of stability) were used before and after the treatment intervention; (3) Results: We found significant differences for the FSA, LOS between pre-test and post-test in the 2 groups; (4) Conclusions: The findings of this study suggest that virtual reality exercise program can be applied as a useful approach for spinal cord injury patients.

## 1. Introduction

Spinal cord injury is caused by diseases or trauma of spinal nerve tissue. According to the 2015 Needs Survey, although spinal cord injury could result from disease (9.8%), its main cause was trauma (89.4%), such as traffic accidents, falls, plunges, and industrial accidents [1]. It has been reported that among the quadriplegic patients with cervical spinal cord injury, 39.5% had incomplete paralysis and 16.3% had complete paralysis, while among the paraplegic patients with spinal cord injury at the T2 nerve or below, 21.7% had incomplete paralysis and 22.1% had complete paralysis [2].

In particular, loss of motor and sensory functions and muscle weakness associated with spinal cord injury impair patient’s sitting postural balance ability [3]. The sitting balance ability in patients with spinal cord injury is an important goal of rehabilitation because it plays an important role in maintaining an independent daily life [4]. Recently, various virtual reality-based methods that enable patients to perform tasks according to their rehabilitation purpose have been introduced to increase the patient’s interest and participation in the treatment and to enhance their functional activity [5]. In virtual reality, the user physically reacts while performing a given task, such as moving or manipulating an object [6]. Visual feedback from virtual reality integrates the senses of subjects whose vestibular sensation, somatosensory, and visual information are mixed up or it can be used as sensory exercise training to improve the subject’s static and dynamic posture control, thereby enhancing their trunk control and balance ability [7]. Several studies have reported the benefits of virtual reality therapy; intensive sensory input around the damaged spinal cord using the patient’s visual and auditory feedback from the virtual reality system along with stimulation on the sensory motor cortex was shown to help restore the damaged spinal cord and improve their balance activity [8,9]. In addition, game-based virtual reality training was shown to increase patient’s concentration and motivation by arousing their interest in treatment and to help the patient recognize the degree of their performance through accurate and rapid sensory feedback on the given tasks, enabling them to control their movement effectively [10]. As described above, there is a possibility that the virtual reality system can improve the balance ability of patients with spinal cord injury, but to date, there are few cases linking sitting balance ability of patients with spinal cord injury with the application of virtual reality system. Therefore, this study aimed to investigate the effects of virtual reality-based balance training program on the sitting balance ability of patients with spinal cord injury.

## 2. Methods

### 2.1. Participants

The criteria for selection were as follows: (1) Those who were diagnosed with paraplegia due to thoracic or lumbar spine injury more than 6 months ago. (2) Those who were categorized as grades C and D of the standard neurological classification scale presented by the ASIA (American Spinal Injury Association). (3) Those who can be maintained Independent sitting position for more than 30 s. The criteria for exclusion were as follows: (1) Those with hearing or visual impairment according to the medical record. (2) Those with musculoskeletal diseases that might affect the experiment. (3) Those who failed to complete the study schedule due to the subject’s personal reason. Ten subjects were recruited per group from rehabilitation centers belonging to the Daegu medical center.

Among the 21 recruited patients, 1 patient were excluded due to sudden deterioration in his health condition. The remaining 20 patients were randomly assigned using computer generated table of random numbers to an experimental group that will receive VR therapy (*n* = 10), and a control group that will receive general occupational therapy (*n* = 10). The general characteristics of the subjects are shown in Table 1. All participants provided informed consent prior to taking part in the study, and all experimental procedures were approved by the Institutional Review Board of the Daegu University.

### 2.2. Procedure and Interventions

This study was designed as a single-blinded, randomized controlled study. A single blind method was applied to eliminate the error by not knowing the subject’s treatment. The experimental and control groups received general occupational therapy consisting of five 30-min sessions per week for 8 weeks. The experimental group received additional 30 min of VR balance training, while the control group received additional 30 min of general rehabilitation during each session over the same time period. General rehabilitation therapy comprised for improving sitting balance anterior weight shifting on sitting balance training [11], Balance Exercise on the Unstable Surfaces [12], reaching task training on sitting position for spinal cord injury [13].

**Figure 1:**
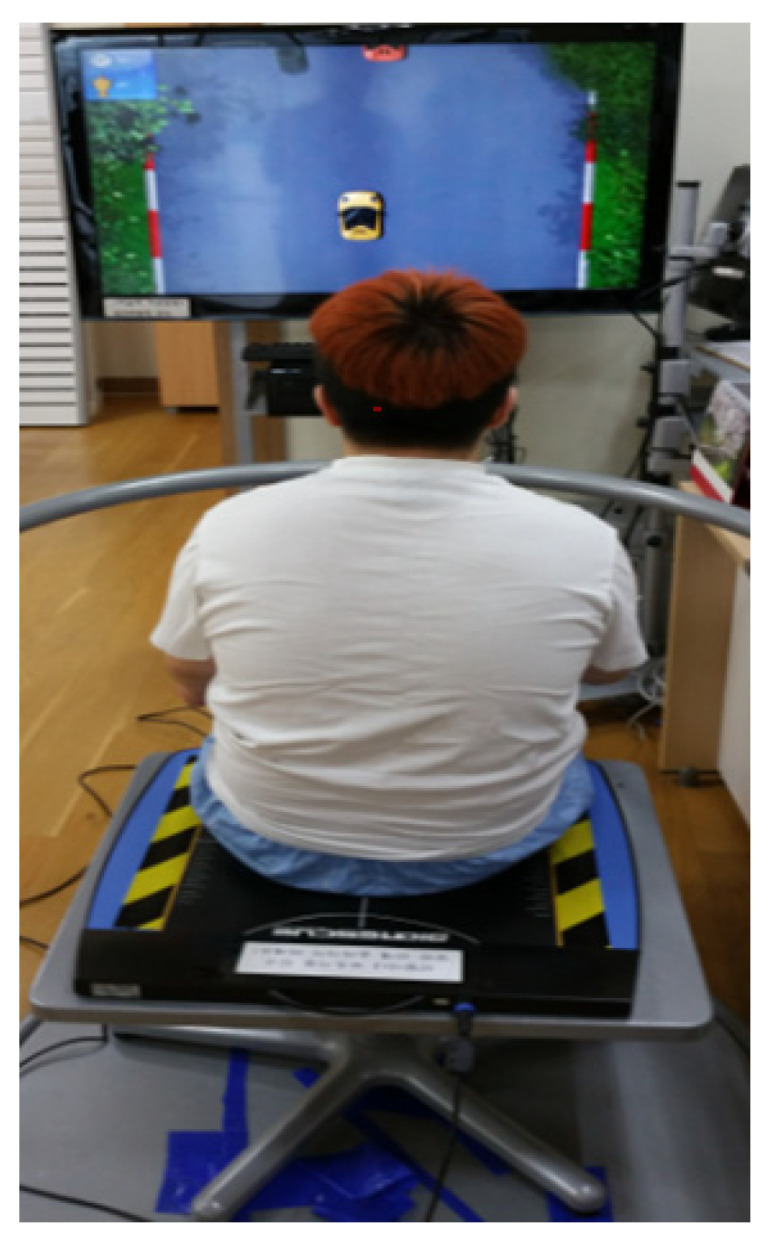
BioRescue system.

The Bio Rescue (RM Ingenierie, Rodez, France), which was the virtual reality device used in this study for the static and dynamic balance, composed of a platform, software, and monitor. The platform (610 mm × 580 mm × 10 mm) is very thin, and is equipped with approximately 1600 pressure sensors. The platform safely and accurately measures the length and area of the center of pressure (COP) in both buttocks of an individual and then transmits the measured data to the computer software. COP is the outcome of the inertial forces of the body and restoring equilibrium forces of the postural control system. Subjects can move freely in the real world while manipulating the virtual objects in a 3D virtual world. To improve the symmetry of weight-bearing, movement, and left/right sitting balance of patients, a total of 3 functional movement programs were applied—the “rally driving”, “air balloon”, “downhill ski”. The degree of difficulty in the movement program varied from stage 1, very easy, to stage 5, very difficult, which depended on the condition of patients. Patients received appropriate audiovisual feedback from the monitor and speaker while performing the exercise game. In this study, the program was administered with the patient sitting on the platform with the monitor placed at 1–1.5 m in front of him. Three types of movement programs, which were appropriate for the condition of each patient, were applied for 10 min each, for a total of 30 min (Figure 1).

### 2.3. Measurements

Assessments were made by an experienced occupational therapist who was blinded to the group assignment and did not take part in the general rehabilitation therapy. Sitting balance ability was measured by Force Sensitive Application and Limit of Stability.

FSA (FSA, Vistamedical, Winnipeg, MB, Canada) comprises of a seat-sized pressure-sensing mat that contains 256 individual piezo-resistive sensors in a 16 × 16 grid. The changes in resistance which result from the different pressures on the sensors are interpreted by the interface module which is connected to a laptop running the FSA software. Data representing the sensor pressure can be displayed as colour-coded maps of pressure distribution, 3-dimensional grids and numeric output parameters. Pressure was measured by comparing the mean pressure distribution value of the left and right of the ischial tuberosity area of 3 × 3 cm around the center of pressure [14].

Dynamic balance was examined using the LOS test with the BioRescue system (RM Ingenierie, Rodez, France). In the LOS test, the subjects moved the COP in 8 directions (left, right, front, back, left front, left back, right front, and right back) to the furthest possible extent while they stood on the support base. The sway area generated by the COP was divided into the surface areas of the 4 directions (left, right, front, and back) and entire area [15,16]. Data collection is the average of three repeated measurements. Three minutes of rest was allowed after every set.

### 2.4. Statistical Analysis

The collected data were analyzed using SPSS version 21.0 (IBM Corp., Armonk, NY, USA). Independent t-test was used for comparison of initial pre-treatment FSA and LOS scores between the two groups. Paired t-test was performed for comparison between the experimental and control groups with respect to changes in pre- to post-treatment FSA and LOS scores, while an Independent t-test was performed for comparison of the magnitude of change between the experimental and control groups. *p* value ˂ 0.05 was considered statistically significant.

## 3. Results

There was no significant difference between the experimental and control groups with respect to pre-treatment FSA and LOS scores (*p* > 0.05; Table 2). Both the experimental and control groups showed a statistically significant increase in post-treatment FSA and LOS scores as compared to the pre-treatment scores (*p* < 0.05; Table 3). Intergroup comparisons showed a statistically significant increase in scores of all assessments in the experimental group as compared to the control group (*p* < 0.05; Table 4). 

## 4. Discussion

For patients with spinal cord injury, restoring their normal sitting posture is one of the factors associated with satisfaction with their quality of life and is also one of the main goals for these patients [17]. Lowered sitting balance ability not only can induce pressure sores and torso malformations, but also can interfere with independent daily life [18].

Patients with spinal cord injury are recommended to use their remaining sensory and motor skills to restore the individual’s new dynamic and static balance abilities [19]. In this study, virtual reality game training that can induce torso movement and stability was applied in order to restore the balance ability of individual patients with spinal injury, and then the effects of such training on the patient’s static and dynamic balance abilities in a sitting posture were investigated.

As the level of balance ability varies depending on the level or extent of injury of each patient with spinal cord injury, the importance of evaluation tools that can clearly and accurately assess the functional state of patients is being emphasized more than ever. The FSA evaluation method can objectively measure the pressure distribution and the changes in pressure center movement on the hips and thighs [14]. Through measuring pressure on the hip, locations of uneven pressure distribution can be identified, and then the cause of scoliosis and the balance ability of the body can be determined. When greater pressure is applied to the tuberosity of the hipbone, the center of pressure for normal people is located in the front, while for patients with a physical disability, it moves backward compared to that of normal people. The limit of stability test(LOS) is a method of measuring total travel distance while the subject moves their body weight in eight directions (forward, backward, left, right, and diagonal) using a decompression platform, which is a BioRescue device, to evaluate their static balance [16]. This method has been utilized as a useful tool for evaluating sitting posture balance in patients with spinal cord injury, because it can measure objective changes numerically. The FSA and LOS evaluation tools are considered more objective than other evaluation tools in that they can measure exact numerical values using computer software.

The virtual reality training program applied in this study required torso stability and delicate movement rather than fast and muscular movement, so this program was found to be effective in improving static and dynamic balance abilities [20]. In our study, statistically significant improvements were seen in post-treatment FSA and LOS scores when compared with the pre-treatment scores. In addition, the experimental group showed a statistically improvement in the balance function when compared with the control group. This is consistent with the results of Kizony et al. who reported that when a virtual reality game program was applied to 13 patients with spinal cord injury in a sitting position, their sitting balance ability was improved [21]. Khurana et al. also reported that after a four-week game-based virtual reality training, the sitting balance ability of paraplegic patients with spinal cord injuries at T6 and T12 levels was better than that of their counterparts who received task-oriented training, which supports the results of the present study [22]. Roopchand et al. reported that after a six-week Wii sport training in patients with spinal cord injuries at ASIA A neurological levels T4 and T12, the distance of their forward arm extension increased, which was an indicative of improvement in their dynamic balance ability [23]. Sengupta et al. reported that game-based VR training program improved trunk postural control in patients with spinal cord injury [24].

Taken together, the combination of virtual reality training and conventional rehabilitation therapy will help to improve the sitting balance ability of patients with spinal cord injury and will become an important intervention method for treating patients with spinal cord injury. This study has limitations in that it is difficult to generalize the result values obtained from this study due to the mall sample size and the difference in performance caused by different injury sites or individual balance ability was not considered in detail. In the future, more research is necessary to supplement these limitations by including more subjects and evaluating individual subjects according to their level of neurological damage in order to find more personalized programs that match the level of need for each patient.

## 5. Conclusions

The results of this study showed that when VR therapy providing functional tasks to the lower extremity was co-administered with general rehabilitation therapy, greater improvement in lower extremity function was seen in spinal cord injury patients, as compared to administering general rehabilitation therapy alone. Therefore, VR therapy combined with standard rehabilitation therapy might be an ideal rehabilitation tool for improving lower extremity function in spinal cord injury patients.

## Figures and Tables

**Table 1:** Characteristics of subjects.

General Characteristics	Division	Virtual Reality Therapy Group (*n* = 10)	Control Group (*n* = 10)	*p*
Sex	Male	9(90.0)	4(40.0)	0.061
	Female	1(10.0)	6(60.0)	
Age (years)		55.1 ± 10.41	53.7 ± 6.55	0.341
Lesion type	Thoracic Spine Injury	10(66.7)	11(73.3)	0.690
	Lumbar Spine Injury	5(33.3)	4(26.7)	
ASIA Impairment Scale	C	7(70.0)	6(60.0)	1.00
	D	3(30.0)	4(40.0)	
Time since SCI		16.5 ± 4.66	17.4 ± 5.12	0.414

ASIA: American Spinal Injury Association.

**Table 2:** Comparison of the baseline in the two groups.

	Mean ± SD		*p*
	Virtual Reality Therapy Group	Control Group	
FSA Left (mmHg)	107.00 ± 12.75	110.47 ± 12.56	0.548
FSA Right (mmHg)	126.44 ± 12.33	115.66 ± 11.59	0.059
LOS (mm^2^)	2784.00 ± 1720.65	2460.00 ± 1571.18	0.665

SD: standard deviation. FSA: force sensitive application, LOS: limit of stability.

**Table 3:** Changes in parameters before and after treatment.

	Virtual Reality Therapy Group (Mean ± SD)			CONTROL Group (Mean ± SD)		
	Before Treatment	After Treatment	*p*	Before Treatment	After Treatment	*p*
FSA Left (mmHg)	107.00 ± 12.75	127.99 ± 8.58	0.001 **	110.47 ± 12.56	118.40 ±10.25	0.011 *
FSA Right (mmHg)	126.44 ± 12.33	136.36 ± 16.06	0.037 *	115.66 ± 11.59	123.20 ± 5.92	0.067 *
LOS (mm^2^)	2784.00 ± 1720.65	5562.60 ± 2129.70	0.000 **	2460.00 ± 1571.18	3632.20 ± 1929.87	0.001 **

SD: standard deviation. FSA: force sensitive application, LOS: limit of stability, * *p* < 0.05, ** *p* < 0.01.

**Table 4:** Comparison of the differences after treatment in the two groups.

	Mean ± SD		*p*
	Virtual Reality Therapy Group	Control Group	
FSA Left (mmHg)	20.99 ± 13.92	7.93 ± 7.90	0.036 *
FSA Right (mmHg)	9.92 ± 12.84	7.54 ± 11.44	0.026 *
LOS (unit: mm^2^)	2778.60 ± 914.30	1172.20 ± 734.62	0.048 *

SD: standard deviation. FSA: force sensitive application, LOS: limit of stability, * *p* < 0.05, ** *p* < 0.01.

## Data Availability

Not applicable.
